# A Widely Metabolomic Analysis Revealed Metabolic Alterations of *Epimedium Pubescens* Leaves at Different Growth Stages

**DOI:** 10.3390/molecules25010137

**Published:** 2019-12-29

**Authors:** Zhenxian Qin, Dengqun Liao, Yalan Chen, Chenyang Zhang, Ruipeng An, Qing Zeng, Xian’en Li

**Affiliations:** Institute of Medicinal Plant Development, Chinese Academy of Medical Sciences and Peking Union Medical College, Beijing 100193, China; qinzhenxian@126.com (Z.Q.); dqliao@implad.ac.cn (D.L.); yalanchen_2017@163.com (Y.C.); zhangchenyang0308@163.com (C.Z.); ruipengan94@gmail.com (R.A.); QingZ_mail@163.com (Q.Z.)

**Keywords:** *Epimedium pubescens* Maxim., flavonoid content, LC-MS/MS, widely targeted metabolome, UPLC-ESI-MS/MS, growth period

## Abstract

*Epimedium* folium is the major medicinally-used organ of *Epimedium* species and its metabolic changes during the leaf growth have not been studied at the metabolomic level. *E. pubescens* is one of five recorded species in the Pharmacopoeia of the People’s Republic of China and widely grows in China. A UPLC-ESI-MS/MS-based targeted metabolomic analysis was implemented to explore the metabolite composition in *E. pubescens* leaves under the cultivation condition and further to investigate their temporal variations among four representative growth stages. A total of 403 metabolites, including 32 hitherto known in *Epimedium* species, were identified in *E. pubescens* leaf, of which 302 metabolites showed the growth/development-dependent alterations. Flavonoid-type compounds were the major composition of the metabolites identified in this study. Most flavonoids, together with tannin-type and lignans and coumarin-type compounds, were up-regulated with *E. pubescens* leaf growth and maturation after the full flowering stage. Our results not only greatly enriched the existing *Epimedium* phytochemical composition database and also, for the first time, provided the metabolomics-wide information on metabolic changes during *E. pubescens* leaf growth and development, which would facilitate in the choice of an optimum harvest time to balance a higher biomass yield of *Epimedium* folium with its better medicinal quality.

## 1. Introduction

*Epimedium* species in *Epimedium* Linn. (Berberidaceace) are perennial shade herbal plants and widely distributed in the world including China, Japan, Korean, North Africa, Europe, and India [1]. Till now, a total of 81 species have been identified in *Epimedium* Linn. (Berberidaceace family), according to the recent NCBI taxonomy database, and more than half of them were found in China (http://www.iplant.cn). *Epimedium* plants are believed to nourish the kidney and reinforce the Yang and used in clinic to effectively treat sexual dysfunction, osteoporosis, cardiovascular diseases, menstrual irregularity, asthma, chronic nephritis, and immunoregulation [1,2]. These wide-reaching pharmacological effects are closely related to the compounds which are synthesized in the growing *Epimedium* plants and accumulated in medicinally used parts especially at harvest. In the recent 40 years, a total of more than 260 compounds have been identified from 20 known species of *Epimedium* Linn., of which were mainly flavonoids [1,2]. However, some of these known metabolites were not distributed in all the *Epimedium* species and showed species-variation or species-specificity [2]. Shi et al. identified 76 essential oils in *E. pubescens* and *E. koreanum* Nakai using GC-MS analysis and found that only 28 of them were shared by these two species [3]. In addition, the collective metabolite constituents in *Epimedium* folium, the major medicinal organ of *Epimedium* species has not to be explored enough in terms of a single *Epimedium* species, considering the estimate that a single plant species can produce about 4000 to 20,000 metabolites [4]. The first three *Epimedium* species with the most number of known chemical compounds were *E. koreanum* Nakai (97), *E. sagittatum* (67), and *E. grandiflorum* (57) [1]. *E. pubescens* is one of five recorded *Epimedium* species including *E. brevicornu* Maxim, *E. sagittatum* Maxim, *E. koreanum* Nakai and *E. wushanense* T.S.Ying in the Pharmacopoeia of the People’s Republic of China (version 2015) [5]. Besides the above-mentioned essential oils, 23 flavonoids were by far identified in this species [6].

Emerging metabolomics provides a large-scale metabolite analysis to mine a matrix of phytochemical compounds presented in given biological samples and to broaden our comprehensive understanding of dynamic changes and developmental regulation of plant metabolism during growth [7,8,9,10]. The primary high-throughput analytical techniques adopted in metabolomic studies include nuclear magnetic resonance (NMR) spectroscopy, gas chromatography-mass spectrometry (GC-MS), and liquid chromatography-mass spectrometry (LC-MS) together with multivariate statistical analysis. Due to its wide metabolome coverage, high sensitivity and accuracy, LC-MS has been greatly used in medicinal plant metabolomics to analyze a wide range of compounds such as flavonoids, alkaloids, terpenoids, steroids, coumarins, and lignans [11]. It also has gradually been used to characterize the spatio–temporal distribution of medicinal plant metabolites at the metabolomic level [12,13,14]. According to the aim and scope of the undergoing research, metabolomics experiments are generally classified into two types: Untargeted metabolomics analysis and targeted metabolomics analysis [15,16]. The former aims to analyze all the measurable known/unknown metabolites in a sample and has the opportunity for novel compound discovery in the studied organism in a specific condition, which most medicinal plant chemists especially long for in their drug discovery projects. In contrast, targeted metabolomics intends to study the metabolites with known chemical structure and biochemically annotated compounds and thus could only find a limited number of known metabolites in the given sample. However, it had an overall better precision in compound identification than the untargeted metabolomics method [17]. In addition, the metabolite coverage and throughput of targeted metabolomics analysis was greatly enhanced with the recent breakthroughs of LC-MS based large-scale targeted metabolomics methodologies and also with the MS/MS library databases constructed and enriched [18,19,20]. Following the widely targeted metabolomics method that Chen et al. developed in 2013 [18], 661 metabolites in *Chrysanthemum morifolium*. cv “Hangju” and 515 metabolites in developing leaves of *Eucommia ulmoides* could be simultaneously identified and quantified using UPLC-ESI-Q TRAP-MS/MS platform in combination of multiple reaction monitoring (MRM) scan mode [13,14].

With the increasing demand on the medicinally-used *Epimedium* species, wild resources are gradually exhausted and in shortage due to over-exploration within short time. People turn to cultivate *Epimedium* plants in China in a simulated shade environment. In this study, *E. pubescens* Maxim. was cultivated in a shaded field and the leaves collected at different growth stages were subjected to flavonoid content quantification and a widely-targeted metabolome profiling. Our result identified/annotated 403 metabolites present in *E. pubescens* leaf, which greatly enriched our current knowledge of compound composition in *Epimedium* metabolism [1,2]. Meantime, our metabolome-based temporal profiling, as the first case in *Epimedium* species research field, revealed that *E. pubescens* leaf possessed huge metabolic changes during the growth, which provided the important and supportive information to choose an optimum harvest time in order to obtain better integrative pharmacological effects of *Epimedium* leaves.

## 2. Results

### 2.1. E. Pubescens Growth in the Shaded Field

**Figure 1:**
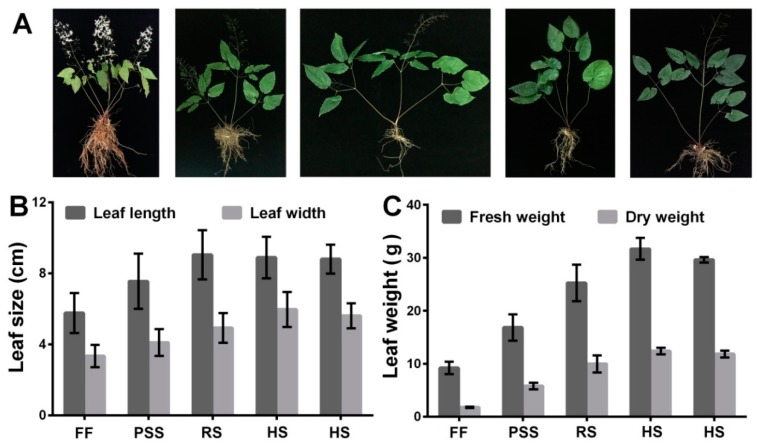
Developmental events of *E. pubescens* plant (**A**) and changes of *E. pubescens* leaf size (**B**) and biomass weight (**C**) at five growth stages: Full flowering stage (FF), peak seed set stage (PSS), ripening stage (RS), and two timepoints of harvest stage (HS). The corresponding observations were made separately on 16 April, 10 May, 3 June, 27 June, and 21 July. Two investigations for harvest stage were carried out on 27 June and 21 July since *E. Pubescens* leaves are harvested usually in June by farmers and sometimes till July, depending on the actual growth status and place of *E. Pubescens* plants.

In our shaded field condition, *E. pubescens* seedlings with tender leaves emerged by the end of February and early March. Figure 1A outlined the major developmental events of *E. pubescens* plants from the full-flowering stage to the mid July. Flowering occurred from March and peaked in middle April. Fruiting simultaneously accompanied the flowering process and peaked in early May. Most follicles matured around early June and fell from the stalks shortly after ripening. *Epimedium* leaves are its major medicinal source. *E. Pubescens* leaves became hard and darker with leaf growth and maturation. Our observation showed that *E. Pubescens* leaf length increased mainly in April and May and reached about 7 cm and that its width stopped increasing till early June (Figure 1B). *E. Pubescens* leaf biomass weight increased with leaf growth and reached the peak by the end of June (Figure 1C), which coincided approximately with the harvesting period in production. In Longnan city, Gansu province where our *E. pubescens* plants were introduced, harvest begins from late May and ends by the end of June.

### 2.2. Flavonoid Content Fluctuated with E. Pubescens Leaf Growth and Development

**Figure 2:**
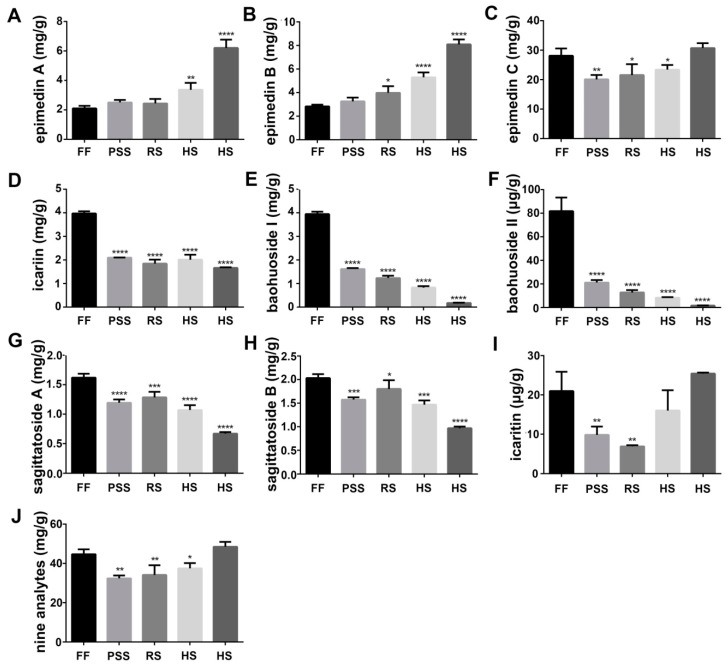
Quantitation results of nine icariin analogues in *E. pubescens* leaves at five growth stages: Full flowering stage (FF), peak seed set stage (PSS), ripening stage (RS), and two timepoints of harvest stage (HS). The observation dates were the same as described in Figure 1. * *p* < 0.05 and ** *p* < 0.01, *** *p* < 0.001 and **** *p* < 0.0001 meant significant difference of the measurement at the indicated stage compared with *E. pubescens* leaf at FF stage. The contents were calculated per gram dry weight. (**A**) epimedin A; (**B**) epimedin B; (**C**) epimedin C; (**D**)icariin; (**E**) baohuoside I; (**F**) baohuoside I; (**G**) sagittatosides A; (**H**) sagittatosides B; (**I**) icaritin; (**J**) total content of nine analytes.

Flavonoids especially prenyl-flavonoids such as epimedin A, epimedin B, epimedin C, and icariin are considered to be the primary bioactive components. They are often used to evaluate the quality of *Epimedium* folium and to study the quality influencing factors such as different growing places, process methods and harvest time. Flavonoid content and composition in *Epimedium* folium were affected by the intrinsic genetic characteristics [21] and environmental factors such as geographical origin [22] and light intensity [23]. In addition, icariine-based content analysis indicated the involvement of developmental regulation on *Epimedium* foliar flavonoid content during the growth [24,25,26,27,28,29]. In this study, we assayed the contents of nine major prenyl-flavonoids in *E. pubescens* leaves using LC-MS/MS method and investigated their dynamic changes with *E. pubescens* leaf growth and development in the simulated shaded field. The representative extraction MRM chromatograms (XIC) for reference standards in negative ion mode were shown in Figure S1. Our results showed that *E. pubescens* leaves contained a less amount of baohuoside II and icaritin (μg/g) and a big amount of epimedins A, B, C, icariin, baohuoside I, and sagittatosides A and B (mg/g) (Figure 2). Their contents fluctuated with *E. pubescens* leaf growth and maturation and displayed two major distinct dynamic changing trends. The first changing trend included epimedin A and epimedin B, which were gradually increased and accumulated mainly after reproductive stages, especially at harvest stage (Figure 2A,B). The content of epimedin A and epimedin B reached 6.21 mg/g and 8.09 mg/g in mid July. The rest seven prenyl-flavonoids had the opposite changing trend from epimedin A and epimedin B. That is, these seven prenyl-flavonoids especially baohuoside I and II were generally higher at the full flowering stage and then decreased greatly at later growth stages. For example, the content of baohuoside I and baohuoside II was 3.95 mg/g and 81.77 ug/g at the full flowering stage and only 0.18 mg/g and 1.59 ug/g in mid July (Figure 2E,F). Some prenyl-flavonoids in this group like epimedin C and icaritin were increased again at late harvest stage and had the close or even higher level, compared to their levels at the full flowering stage (Figure 2C,I). The total contents of these nine prenyl-flavonoids were also found highly accumulated at flowering stage and harvest stage (Figure 2J). Their total content declined to some degree at fruit stages.

### 2.3. Widely-Targeted Metabolomics Analyis of E. Pubescens Leaves at Various Developmental Stages

#### 2.3.1. Overview of *E. Pubescens* Foliar Metabolome

According to our above investigation on *E. pubescens* leaf indexes and developmental changes of flavonoid content, we conducted a targeted metabolomics analysis on the first four representative developmental stages of *E. pubescens* leaves. The accuracy and reproducibility of metabolite detection was revealed by the superimposed display analysis of mass spectrometry total ion current (TIC) of foliar QC samples which were run at the different time (Figure S2A,B). The TICs were well-overlapped, indicating our instrumental stability during the analysis. A total of 403 metabolites, including 216 metabolites detected in the positive ion mode and 187 metabolites detected in the negative ion mode, were identified in *E. pubescens* leaf samples using UPLC-ESI-Q TRAP-MS/MS method (Table S1). The identified metabolites were comprised of 138 flavonoids, 52 lipids, 48 phenolic acids, 46 amino acids and its derivatives, 26 nucleotide and its derivatives, 23 organic acids, 21 alkaloids, 7 tannins, 17 saccharides and alcohols, 7 vitamins, 5 lignans and 1 coumarins, 2 quinones, and 10 other metabolites. The flavonoids included 57 flavonols, 46 flavonoids, 11 flavanols, 9 dihydroflavones, 5 anthocyanins, 4 dihydroflavonols, 3 chalcones, 3 isoflavones, and 1 other type. Of 403 identified metabolites, 158 metabolites were annotated in KEGG compound database and 127 were mapped to 94 KEGG pathways (3rd level).

#### 2.3.2. Multivariate Analysis of *E. Pubescens* Foliar Metabolomes at Various Developmental Stages

**Figure 3:**
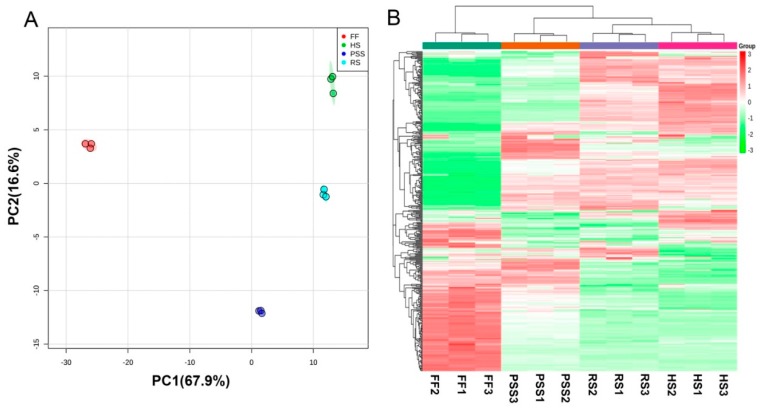
Score plot of principal components analysis (PCA) (**A**) and heatmap of hierarchical cluster analysis (HCA) (**B**) showing that the leaf development regulated *E. Pubescens* foliar metabolites. The abbreviated stage name and observation dates were the same as described in Figure 1. HS in metabolomic analysis referred to leaf samples collected on 27 June.

In order to reveal the developmental roles in *E. Pubescens* foliar metabolism, the resulting metabolomic data from four main growth stages were further subjected to multivariate statistical analysis. Unsupervised PCA (Figure 3A) and hierarchical cluster analysis (HCA) heatmap (Figure 3B) showed that the obvious metabolic changes occurred in *E. Pubescens* leaves across the growth and development. The first principal component (PC1) explained 67.9% of the variance, followed by the second principal component (PC2) with 16.6%, which both amounted to a total variance of 84.5%. The samples of four different growth stages were separated from one another. The FF stage was markedly clustered on the negative side of PC1 and far from other three growth stages, while the RS- and HS- samples were grouped on the positive side of PC1. Similarly, the HCA heatmap result clustered FF stage from other three growth stages, indicating a great number of metabolites changed in *E. pubescens* leaf after the full flowering stage. RS- and HS- stage were clustered closer than PSS group, indicating that RS- and HS- stage had more similar metabolite profiling, compared to PSS.

#### 2.3.3. Differential Metabolite Profiling of *E. Pubescens* Leaves during the Leaf Growth and Development.

**Figure 4:**
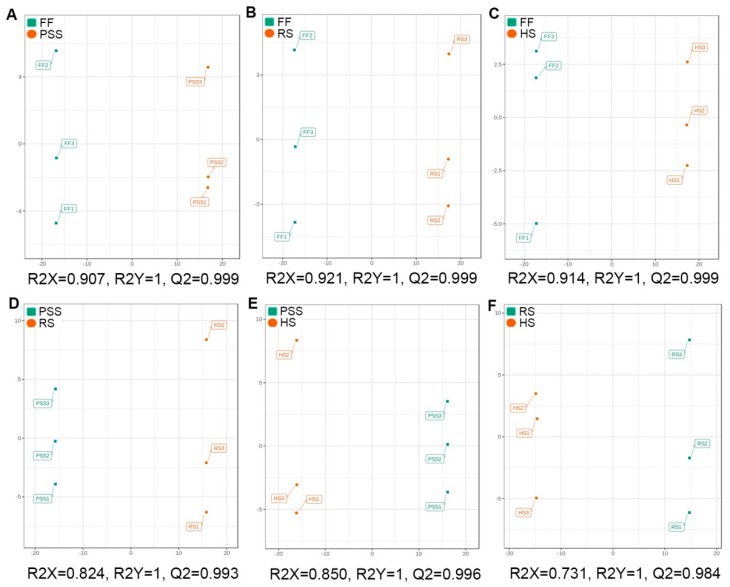
Score plots of orthogonal partial least squares discriminant analysis (OPLS-DA) for six pairwise comparisons. R2X and R2Y were cumulative modeled variation of all R2Xs and R2Ys, separately, and Q2 was the cumulative predicted fraction. The abbreviated stage name and observation dates were the same as described in Figure 3. (**A**) PSS vs FF; (**B**) RS vs FF;(**C**) HS vs FF;(**D**) PSS vs RS;(**E**) PSS vs HS;(**F**) RS vs HS.

In order to find out the differential metabolites regulated by *E. pubescens* leaf growth and development, a supervised OPLS-DA was further conducted to construct the classification models and discriminate the different metabolic compositions between the compared growth stages. As shown in OPLS-DA score plots (Figure 4A–F), clear metabolic differentiations in *E. pubescens* leaves were observed between PSS and FF, RS and FF, HS and FF, RS and PSS, HS and PSS, and HS and RS. The R2X and Q2 values (Figure 4A–F) and permutation tests (Figure S3A–F) suggested that these models had the high fitness and predictive capability and were suitable for further differential metabolite analysis.

**Figure 5:**
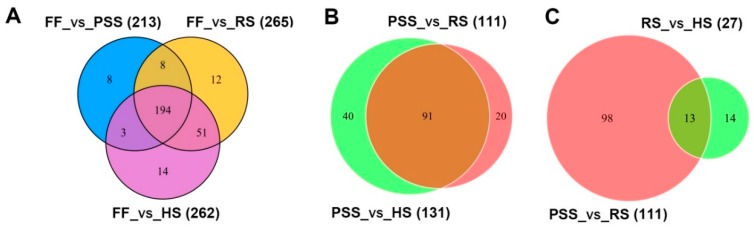
Venn diagrams showing differential metabolites shared or unique among the different comparisons. (**A**) Differential metabolites detected by comparisons of FF stage and the remaining three growth stages. (**B**) Differential metabolites detected by comparisons of PSS stage and its subsequent two stages. (**C**) differential metabolites detected by comparisons of RS stage and its two neighboring stages: PSS and HS stages. The number in () was the altered metabolites between two compared growth stages. The numerical values on the Venn diagram depicted the number of differential metabolites shared or unique among the different comparisons. The abbreviated stage name and observation dates were the same as described in Figure 3.

Using two differential metabolite screening criteria VIP ≥ 1 and |Log2FC (fold change)| ≥ 1, a total of 302 metabolites from 403 annotated metabolites were identified to differentially express in at least two studied stages (Table 1 and Table S2). As shown in Table 1, *E. pubescens* leaves at the full flowering stage had the remarkably different contents of metabolites from the subsequent three growth stages. Compared to FF stage, the contents of 213, 262, and 265 metabolites were found changed at PSS, RS, and HS stage, respectively. Venn diagram analysis further revealed that 194 differential metabolites were commonly regulated by three late growth stages and only few compounds were uniquely modulated by a specific growth stage (Figure 5A). We further compared the metabolite contents of *E. pubescens* leaves at the subsequent three stages, namely PSS, RS, and HS stages (Table 1, Figure 5B,C). 111 metabolites (52 increased/59 decreased) in RS stage and 131 metabolites (62 increased/69 decreased) in HS stage were found significantly distinct, compared to PSS stage. There were only 27 differentially regulated metabolites between RS stage and HS stage, which indicated that most metabolites in *E. pubescens* leaves remained a relatively constant abundance with its leaf maturation and senescence. The number of differential metabolites in each compound class was also investigated (Table 1). Our targeted metabolomic data uncovered that a high proportion of metabolites in each compound class were highly regulated by *E. pubescens* leaf growth stage. For example, 119 of 138 flavonoids ~87%, as the major bioactive compound resources of *Epimedium* folium, showed the altered contents during the leaf growth and development.

**Figure 6:**
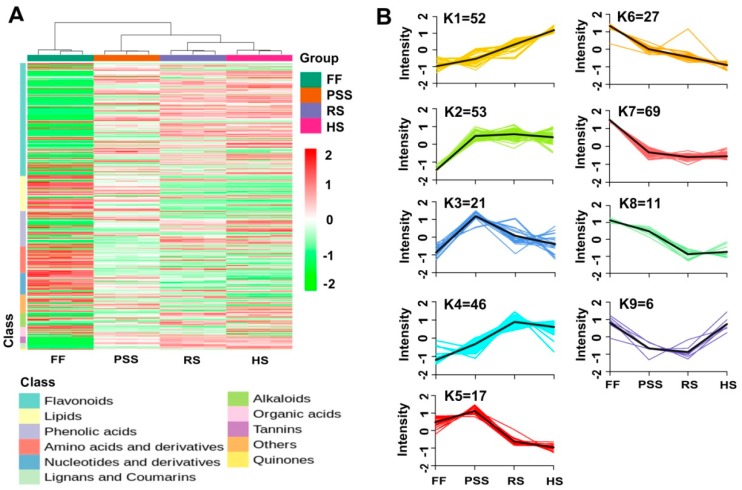
HCA (**A**) and K-means (**B**) cluster analysis showing the dynamic accumulation of 302 differential metabolites across four representative growth stages. K-means cluster was performed on Z-score normalized intensities. The abbreviated stage name and observation dates were the same as described in Figure 3.

The hierarchical and K-means cluster analyses were performed to characterize dynamic changes of differential metabolites in *E. pubescens* leaves across the four studied growth stages (Figure 6A–B). The HCA-based heatmap showed that the four growth stages of leaf samples were clustered into three major groups: FF stage, PPS stage, and RS and HS stages. The HCA also revealed that most flavonoid-type compounds together with tannin-type and lignans and coumarin-type were up-regulated with *E. pubescens* leaf growth and development, while lipid-type and amino acid-type compounds were down-regulated. The k-means clustering further classified all differential metabolites into nine sub-clusters (Figure 6B, Table S2). Sub-clusters 1–5 showed the similar metabolite changing trends, that is, the differential metabolites in these five groups had increased contents in *E. pubescens* leaves after the full flowering stage. Sub-cluster 1 contained 52 metabolites whose contents constantly increased throughout the whole growth period. The representative compounds in this group included flavonoid glycosides astragalin, icariin, luteolin and its metabolites, and phenylpropanoid neochlorogenic acid. Although the abundance of metabolites in Sub-clusters 2 and 3 were dramatically increased at PSS stage, the metabolites in the former sub-cluster remained constant during late stages while in Sub-cluster 3 declined greatly at ripening stage and then remained the slightly higher levels at harvest stage than at the full flowering stage. The metabolites in Sub-clusters 4 and 5 reached its accumulation plateau at the ripening stage and harvest stage, separately and then exhibited the similar changes as Sub-cluster 2 and 3, separately. Sub-clusters 6–9 consisted of metabolites whose levels decreased greatly after the full-flowering stage. 27 metabolites in Sub-cluster 6 and 69 in Sub-cluster 7 reached their lowest levels at the harvest stage and ripening stage, separately. The representative compounds in clusters 6–9 included chlorogenic acid, baohuoside I, lkarisoside D, lkarisoside C, and lipid-type compounds LysoPE 18:1, LysoPC 18:3, LysoPC 16:0, and LysoPE 16:0.

#### 2.3.4. KEGG Pathway Enrichment Analysis of Differential Metabolites

Of 127 metabolites assigned to KEGG pathways (Table S1), 95 exhibited the fluctuating accumulation in *E. pubescens* leaves during the growth (Table S2). Thirty-eight more differential metabolites were putatively assigned onto KEGG pathways according to the similarities of their chemical structures with known KEGG compounds (Table S2). The further KEGG pathway enrichment analysis revealed that only “Flavonoid biosynthesis” was significantly different in the comparisons of FF and PSS, FF and HS, PSS and RS, and PSS and RS (Table S3). This indicated that flavonoid accumulation in *E. pubescens* leaves was tightly regulated by plant development during reproductive stages and maturation.

## 3. Discussion

*Epimedium* folium, named Yingyang huo in Chinese, is its major medicinally-used organ of this plant. As a perennial herb, *Epimedium* leaves experience an annual plant life cycle: emergence, growth and development, and maturation towards senescence. A number of physiologic activities accompanied with dramatic metabolic changes occurred in plant leaves during the year-round plant life cycle [14,30]. The influences of plant growth/development on *Epimedium* foliar constituents were studied on the relative contents of icariine [24,25,26,27,28,29], epimedin A [27], epimedin B [27], epimedin C [25,27] and sagittatoside B [28], and rouhuoside [29]. Although no consistent optimum time for the higher flanovoid content could be concluded from these studies, possibly due to the difference of experimental growth conditions and the studied *Epimedium* species, all these findings told that their contents fluctuated during the whole growth period. They usually had a higher accumulation in young *Epimedium* leaves and declined at the reproductive stage and increased later on to some extent, which was similar as our quantification of icariine and its analogues in *E. pubescens* leaves at the different growth stages. Our targeted metabolomics analysis also elucidated the overall developmental regulation of *Epimedium* foliar ingredients especially flavonoid compounds after the full flowering stages. Most of detected flanovoid compounds such as luteolin, acuminatoside, hyperinsuch, icariin and icaritin were significantly increased with *E. pubescens* leaf growth and development. It should be noted that the other main *Epimedium* bioactive compounds such as epimedins A, B, C, sagittatoside A, and sagittatoside B were just slightly changed in our four studied growth stages where *E. pubescens* leaves were already fully expanded and almost mature especially at harvest time. In *E. sagittatum*, a higher accumulation of main epimedium bioactive compounds epimedin A, B, and icariin was found in folded young leaves, not in expanded mature leaves [31]. KEGG pathway enrichment analysis revealed that flavoniod biosynthesis was significantly influenced by *E. pubescens* leaf growth and development. Transgenic studies showed the synthesis of major bioactive flanovoids was regulated by transcription factors such as MYB9 EsMYBA1, EsMYBF1, and EsGL3 [31,32]. Further expression profiling will facilitate us to understand the molecular mechanisms of developmental dynamic changes of *E. pubescunes* foliar metabolites at crucial growth and development stages.

## 4. Materials and Methods

### 4.1. Chemicals and Reagents

HPLC-grade methanol, ethanol and acetonitrile were purchased from Merck Corporation (Darmstadt, Germany). Analytical grade formic acid and acetic acid were provided by the Tianjin Reagent Company (Tianjin, China). Distilled water was purchased from Wahaha Group Co., Ltd. (Hangzhou, China). Standard compounds of nine icariin analogues including epimedin A, epimedin B, epimedin C, icariin, baohuoside I, baohuoside II, sagittatoside A, sagittatoside B, and icaritin were obtained from Shanghai Yuanye Biotechnology (Shanghai, China). All these standards were HPLC-grade and ≥98% of purity.

### 4.2. Plant Growth and Sampling

*Epimedium* (*Epimedium pubescens* Maxim.) rizhome buds were transplanted in October, 2016 from Longnan City, Gansu province, China to the shade mesh field at the Institute of Medicinal Plant Development (IMPLAD), Beijing, China. The plant and row distances were separately 15 cm and 60 cm. The black shade mesh was fixed onto 1.6 m high steel poles and can filter 80% sunlight irradiation. *Epimedium* leaves were harvested in the morning about every 24 days from 16 April, 2019 till 21 July, 2019. Each time, three biological replicates were collected from multiple uniformly-grown plants. In order to remove leaf surface soil, the collected leaves were rinsed quickly in the tap water and then dried with tissue paper. Subsequently, one portion of leaf samples for widely-targeted metabolomics analysis were flash frozen in liquid nitrogen and then stored at −80 °C till use. The other portion of leaf samples for quantitative analysis of nine icariin analogues were brought to the lab and dried at 60 °C in the oven for 12 h.

*E. pubescens* leaf dynamic growth was investigated using leaf indexes including leaf size (width X length) and leaf fresh and dry weight. Branches with three compound leaflets were collected each time randomly from the field. Leaf width and length of the middle leaflet was measured separately across its widest part and along its major vein. One replicate of leaf weight was measured from 20 branches and three replicates were done each stage. *E. Pubescens* leaf dry weight was measured after constant weight was obtained in the oven at 60 °C. The significant difference of leaf indexes across the studied growth stages (adjusted *p* < 0.05) were evaluated using one-way analysis of variance (ANOVA) with Tukey’s multiple comparisons test in GraphPad Prism 6.0 (La Jolla, CA, USA).

### 4.3. LC-MS/MS Quantitative Analysis of Icariin Analogues

#### 4.3.1. Preparation of Quantitative Analysis Samples and Standard Solutions

The oven-dried leaf samples were powdered by the electric universal pulverizer and sieved via a 60 mesh sieve. Then, 50 mg of each *Epimedium* leaf powder was weighed and extracted in 25 mL of 40% (*v*/*v*) ethanol solution at room temperature for one hour using an ultrasonic extraction system. The extracted solutions were filtered under vacuum and the filtrates was then filtered via a 0.22 μm membrane and transferred into the inner vial for the subsequent analysis.

The appropriate amounts of epimedin A, epimedin B, epimedin C, icariin, baohuoside I, baohuoside II, sagittatoside A, sagittatoside B, and icaritin were separately weighed and dissolved in methanol as individual standard stock solutions. A series of mixed standard solutions were diluted by methanol to the suitable concentrations of 16.2–540 ng/mL for epimedin A, 27.8–555 ng/mL for epimedin B, 0.053–10.6 μg/mL for epimedin C, 10.3–515 ng/mL for icariin, 5.15–386 ng/mL for Baohuoside I, 1.29–645 ng/mL for Baohuoside II, 5.4–405 ng/mL for sagittatoside A, 2.475–495 ng/mL for sagittatoside B and 1.07–53.5 ng/mL for icaritin. All standard stock and work solutions were stored at −20 °C freezer.

#### 4.3.2. LC-MS/MS Analysis

The analysis was conducted on a SHIMADZU Prominence LC system (Kyoto, Japan) coupled with a 5500 QTRAP mass spectrometer (AB SCIEX, Foster City, CA, USA), which includes a LC-20AD_XR_ solvent delivery system, a DGU-20A_3R_ automatic degasser, a SIL-20A_XR_ autosampler and a CTO-20AC column oven. Chromatographic separation was performed at 40 °C on a Waters UPLC BEH C18 column (2.1 mm × 100 mm, 1.7 μm) The mobile phase consisted of a binary solvent system with ACN (A) and 0.1% formic acid in water (B), and run under the following parameters: 35% A at 0.01–0.5 min, 35–90% A at 0.5–9 min, 90% A at 9–11 min, 90–35% A at 11–12 min and 35% of A for 4 min to re-equilibrate the column. The injection volume was 2 μL and the flow rate was set to 0.3 mL/min.

The mass spectrometric detection was operated in the multiple reaction monitoring (MRM) mode with the negative electrospray ionization (ESI). The optimized ESI parameters included ion spray voltage of 4500 V, temperature of 550 °C, curtain gas of 0.24 Mpa, nebulizer gas of 0.38 Mpa and heater gas of 0.38 Mpa. The parameters of MRM transitions, declustering potential (DP) and collision energy (CE) of nine analytes were optimized using a syringe infusion pump and are listed in Table 2. Data acquisition and analysis were processed by the Analyst software (V.1.6.2) from AB SCIEX (Concord, ON, Canada).

#### 4.3.3. Method Validation

The quantitative method in this study was assessed by calculating the parameters linearity, precision, repeatability, stability, limit of detection (LOD), and limit of quantification (LOQ). The calibration curve linearity of each analyte was calculated by plotting the acquired peak areas versus concentrations of this calibration standard. The correlation coefficient (r) was used to estimate the linearity of calibration curves. The limits of detection and quantitation were set at a signal-to-noise ratio of 3:1 and 9:1, separately. The precision was determined by six repeated measurements of a certain concentration of mixed standard solution within a day and expressed by the relative standard deviation (RSD). The repeatability was evaluated by determination of the analytes in six independently prepared extracts of a leaf sample, which was expressed as RSD. To determine the extract stability, the same leaf extract was analyzed at 0, 2, 4, 8, 16, and 24 h at room temperature, respectively. Variations were expressed as RSD. The relevant results of method validation parameters were seen in Table S4 and showed that the established LC-MS/MS method was suitable for quantitative analysis of icariin analogues in *Epimedium* samples. The concentrations of nine flavonoid analytes in *E*. *pubescens* leaf samples were calculated using their corresponding calibration curves, which were constructed using the series working standard solutions. Their absolute content was expressed by the amount per gram leaf dry biomass.

### 4.4. Widely-Targeted Metabolomic Analysis of E. Pubescens Leaves

#### 4.4.1. Leaf Metabolite Extraction

The freeze-dried leaf was crushed into a fine powder using a laboratory mixer mill (MM 400, Retsch) with a zirconia bead for 1.5 min at 30 Hz. 100 mg of leaf powder was weighed and extracted overnight at 4 °C with 1.2 mL of 70% methanol, vortexed for six times and then centrifuged at 10,000 rpm for 10 min. The supernatants were collected and filtered via 0.22 μm membranes and then transferred into the inner vials for UPLC-ESI-MS/MS analysis. Simultaneously, a quality control sample (QC) was prepared by mixing all of the samples.

#### 4.4.2. UPLC- ESI-Q TRAP-MS/MS Analysis of E. Pubescens Leaf Metabolomes

A UPLC system (SHIMADZU, Kyoto, Japan) connected with 6500 QTRAP mass spectrometer (AB SCIEX, Foster City, CA, USA) and equipped with an ESI source was applied to conduct the metabolomic analysis. A Waters ACQUITY UPLC HSS T3 C18 column (2.1 mm × 100 mm, 1.8 μm) was used to separate the metabolites in *E*. *pubescens* extracts. The column temperature maintained at 40 °C. The mobile phase consisted of a binary solvent system with water containing 0.04% acetic acid (A) and ACN containing 0.04% acetic acid (B), and run under the following gradient parameters: 5–95% B at 0–10 min, 95–95% B at 10–11 min, 95–5% B at 11–11.1 min and 5% of B for 2.9 min to re-equilibrate the column. The injection volume was 2 μL and the flow rate was set to 0.35 mL/min. Linear ion trap (LIT) and triple quadrupole (QQQ) scans were acquired on the 6500 QTRAP mass spectrometer in negative and positive ion modes, which was controlled by the Analyst software (V.1.6.3, AB SCIEX). The ESI operating parameters were set as: source temperature, 550 °C; ion spray voltage, 5500 V for the positive ion mode and –4500 V for the negative ion mode; ion source gas I, 50 psi; ion source gas II, 60 psi; curtain gas, 30 psi; the collision gas (CAD) was high. Instrument tuning and mass calibration were performed with 10 and 100 μmol/L polypropylene glycol solutions in QQQ and LIT modes, respectively. The mass spectrometric detection was operated in MRM mode with collision gas (nitrogen) set to 5 psi. DP and CE for individual MRM transitions were acquired by further DP and CE optimization. A specific set of MRM transitions were monitored for each period based on the metabolites eluted within this period [18].

During the analysis, the quality control sample was injected recurrently throughout the analytical run to monitor the UPLC-MS/MS system. *E*. *pubescens* samples were loaded in the random order.

#### 4.4.3. Data Processing, Metabolite Identification and Quantitation and Multivariate Statistical Analysis

The secondary mass spectrum data were acquired and pre-processed using the Analyst software (V.1.6.3, AB SCIEX). The isotope signal, the repeated signals such as K^+^, Na^+^, NH4^+^, and fragments from the high molecular weight metabolites were excluded in the analysis. The characteristic ions of each compound were screened out by the triple quadrupole rod, and their signal intensities were detected in MRM mode. The metabolites in *E*. *pubescens* leaves were determined based on the secondary spectrum information of the Metware database MWDB (Metware Biotechnology Co., Ltd. Wuhan, China). Meantime, the mass spectrometry file was imported to the MultiaQuant software 3.0.3 for peak detection, integration and correction. The corrected peak area of the strongest secondary characteristic ion (cps, count per second) was used to quantify the relative content of the identified metabolite, which was further used for the below multivariate analysis (MVA) and the identification of differential metabolites with *E*. *pubescens* leaf development through the full-flowering stage till the harvest stage.

The peak area data were log2-transformed, mean-centered and unit variance scaled prior to PCA (principal component analysis) and OPLS-DA (orthogonal to partial least squares-discriminate analysis). PCA was performed using the statistics function prcomp within R package (Ver. 3.5.0) (www.r-project.org). OPLS-DA was conducted using R package MetaboAnalystR (Ver. 1.0.1). A permutation test (200 permutations) was performed to validate the fitness of OPLS-DA models. The differentially regulated metabolites between two compared groups were determined by VIP ≥ 1 and |Log2FC (fold change)| ≥ 1. The developmental changing patterns of the differentially regulated metabolites in *E*. *pubescens* leaves were visualized using the hierarchical cluster analysis (HCA) (pheatmap, R Ver. 1.0.12) and K-means cluster.

#### 4.4.4. KEGG Pathway Analysis of the Identified Metabolites

Identified metabolites were annotated using KEGG Compound database and mapped to KEGG Pathway database (http://www.kegg.jp/kegg). KEGG pathway enrichment analysis of differential metabolites was done in MSEA (metabolite sets enrichment analysis). Their significance was determined by the hypergeometric test and was set as the corrected *p* < 0.05.

## Figures and Tables

**Table 1:** Number of differential metabolites found in different pairwise comparisons and compound class.

	Compared Stages	PSS vs. FF		RS vs. FF		HS vs. FF		RS vs. PSS		HS vs. PSS		HS vs. RS	
Compound class		Up	Down	Up	Down	Up	Down	Up	Down	Up	Down	Up	Down
Flavonoids (**119/138**)		84	18	88	22	85	22	26	21	30	22	6	2
Lipids (**37/52**)		6	15	5	29	5	27	2	20	2	21	0	2
Phenolic acids (**37/48**)		16	10	20	12	19	13	10	7	11	8	3	2
Amino acids and its derivatives (**28/46**)		2	14	7	20	5	19	4	3	5	4	0	3
Nucleotide and its derivatives (**23/26**)		3	8	3	12	4	15	1	3	0	6	0	2
Organic acids (**10/23**)		2	3	3	4	3	6	0	2	2	3	0	1
Alkaloids (**15/21**)		6	3	9	3	9	4	4	2	4	2	1	1
Tannins (**7/7**)		7	0	7	0	7	0	1	0	1	0	0	0
Lignans and coumarins (**5/6**)		5	0	5	0	5	0	1	0	2	0	0	0
Quinones (**2/2**)		1	0	1	1	1	1	0	0	0	0	0	0
Other metabolites (**19/34**)		4	6	6	8	6	6	3	1	5	3	3	1
**Significant differentials**		136	77	154	111	149	113	52	59	62	69	13	14
**All Significant differentials (302)**		**213**		**265**		**262**		**111**		**131**		**27**	

Note: the number in (/) behind compound class referred to the total differential metabolites in this class found in all the comparisons and the total number of compounds in the metabolomic data. The class “other metabolites included three subclasses: saccharides and alcohols, vitamins, and others. Up/Down referred to the change trend of the metabolite content in the stage compared to the early stage.

**Table 2:** Multiple reaction monitoring conditions of nine analytes in negative ionization mode.

Analyte	Time (min)	Precursor Ion (*m/z*)	Product Ion (*m/z*)	DP (v)	CE (v)
epimedin A	1.26	883.4	675.3	−92	−30
epimedin B	1.32	853.5	645.3	−100	−27
epimedin C	1.37	867.4	659.3	−110	−24.9
icariin	1.57	721.4	513.3	−120	−23
baohuoside I	4.79	513.3	366.1	−120	−36
baohuoside II	3.37	499.3	352.2	−190	−37.5
sagittatoside A	4.01	675.4	366.2	−180	−45
sagittatoside B	4.26	645.3	366.2	−130	−44
icaritin	7.45	367.3	352.2	−170	−29.3

## Supplementary Materials

Supplementary materials can be found at https://www.mdpi.com/1420-3049/25/1/137/s1. Figure S1. Representative extraction MRM chromatograms (XIC) of nine reference standards in negative ion mode. Figure S2. The overlapped total ion chromatograms of *E. pubescens* QC samples showing the accuracy and reproducibility of metabolite detection operated on our analytic platform. (A) positive ESI mode and (B) negative ESI mode. Figure S3. Permutation tests on OPLS-DA models. The horizontal line represented R2Y and Q2 calculated in the original OPLS-DA model. R2′Y and Q2′ calculated by permutation tests. Table S1 The identified metabolites and their expression profiles in *E. pubescens* leaves. Table S2. List of differential metabolites in *E. pubescens* leaves. Table S3. KEGG enrichment analysis of differential metabolites in six pairwise comparisons. Unique compound and compound referred to the number of the differential compounds assigned to the pathway separately in two compared stages and in all six comparisons. Uni_all and compound_all referred to the total number of differential compounds assigned to all KEGG pathways and the total number of the compounds assigned to all KEGG pathways. Table S4. Method validation for simultaneous quantification of icariin analogues in *E. pubescens* samples.
