# Supplementary material for: A Widely Metabolomic Analysis Revealed Metabolic Alterations of Epimedium Pubescens Leaves at Different Growth Stages
Source: Molecules. 2019 Dec 29;25(1):137. doi: 10.3390/molecules25010137 (PMC6982732; doi:10.3390/molecules25010137)
Supplement: Supplementary file 1 [file molecules-25-00137-s001.pdf]

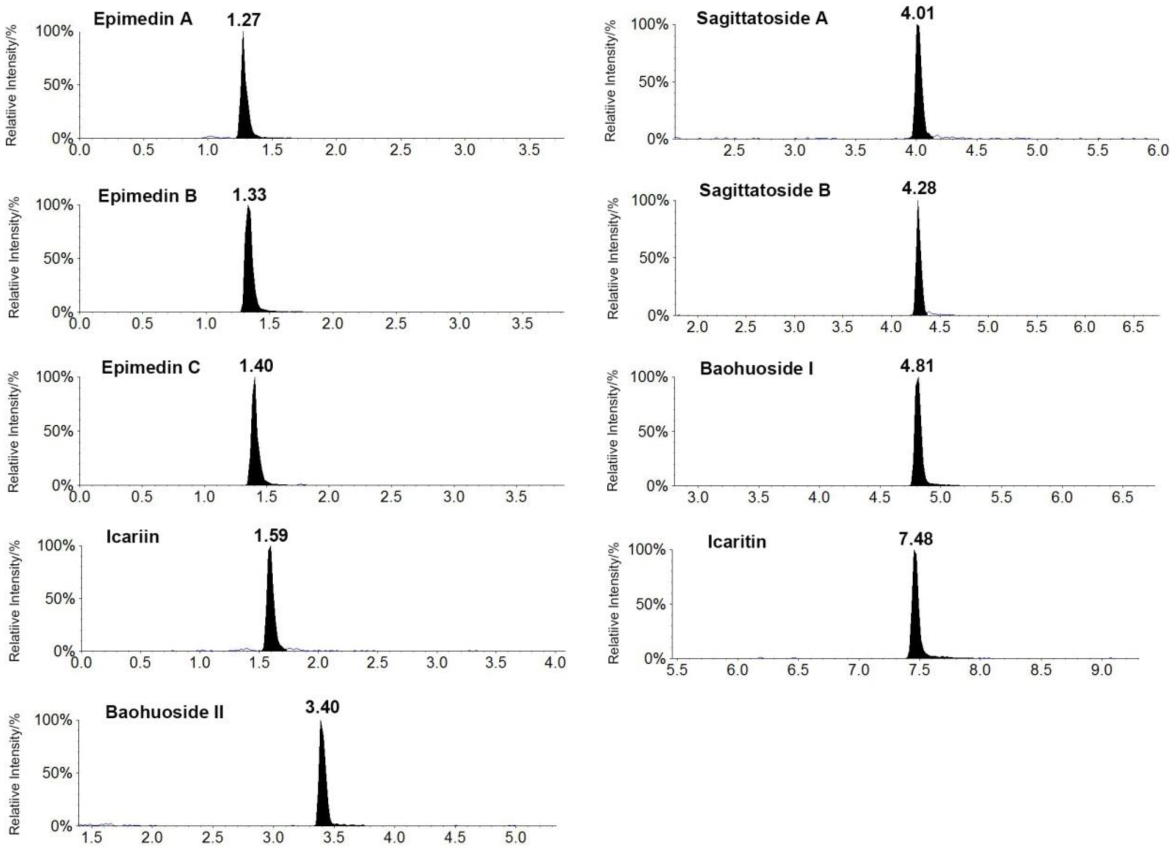

Figure S1. Representative extraction MRM chromatograms (XIC) of nine reference standards in negative ion mode.

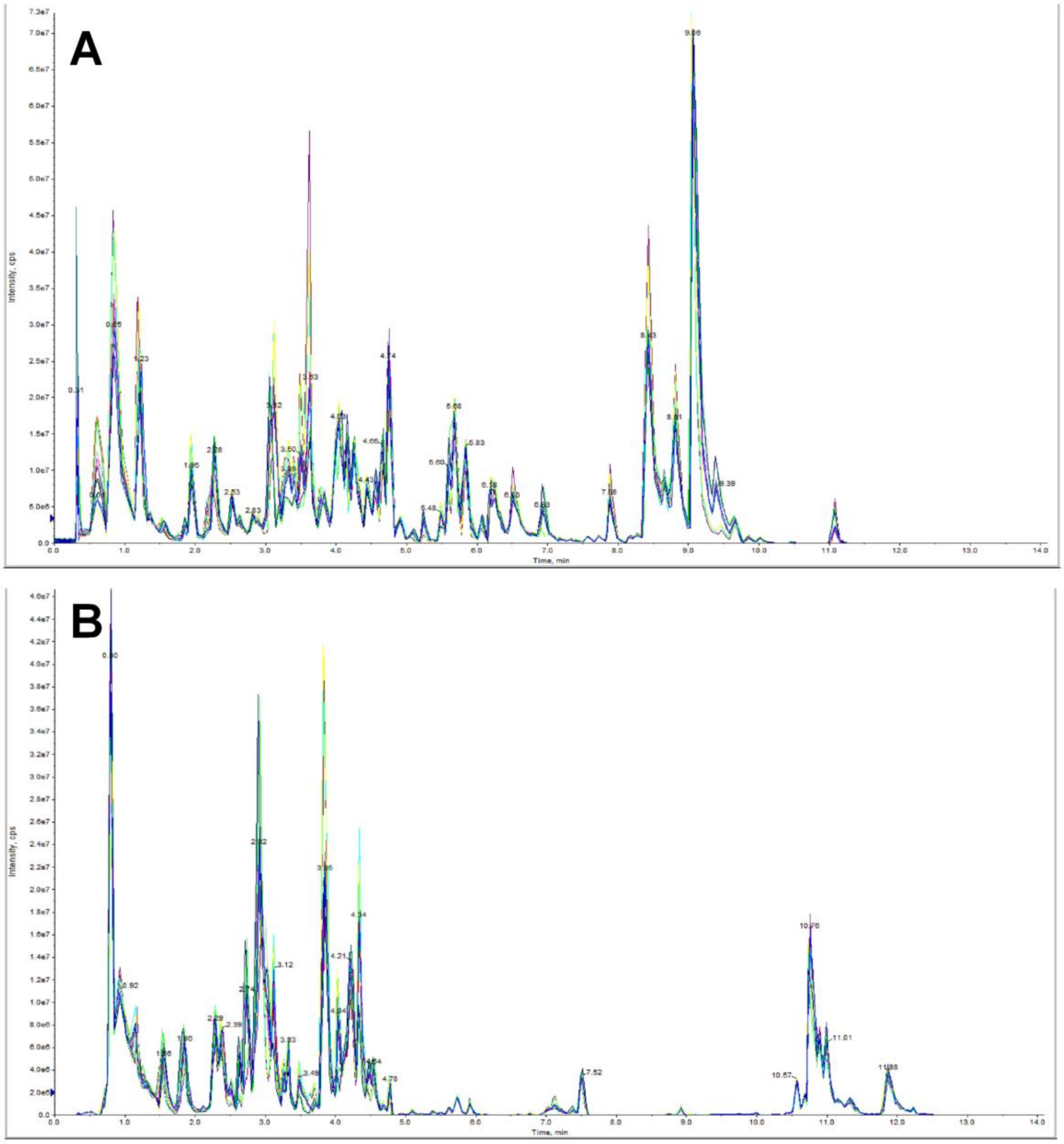

Figure S2. The overlapped total ion chromatograms of *E. pubescens* QC samples showing the accuracy and reproducibility of metabolite detection operated on our analytic platform. (A) positive ESI mode and (B) negative ESI mode.

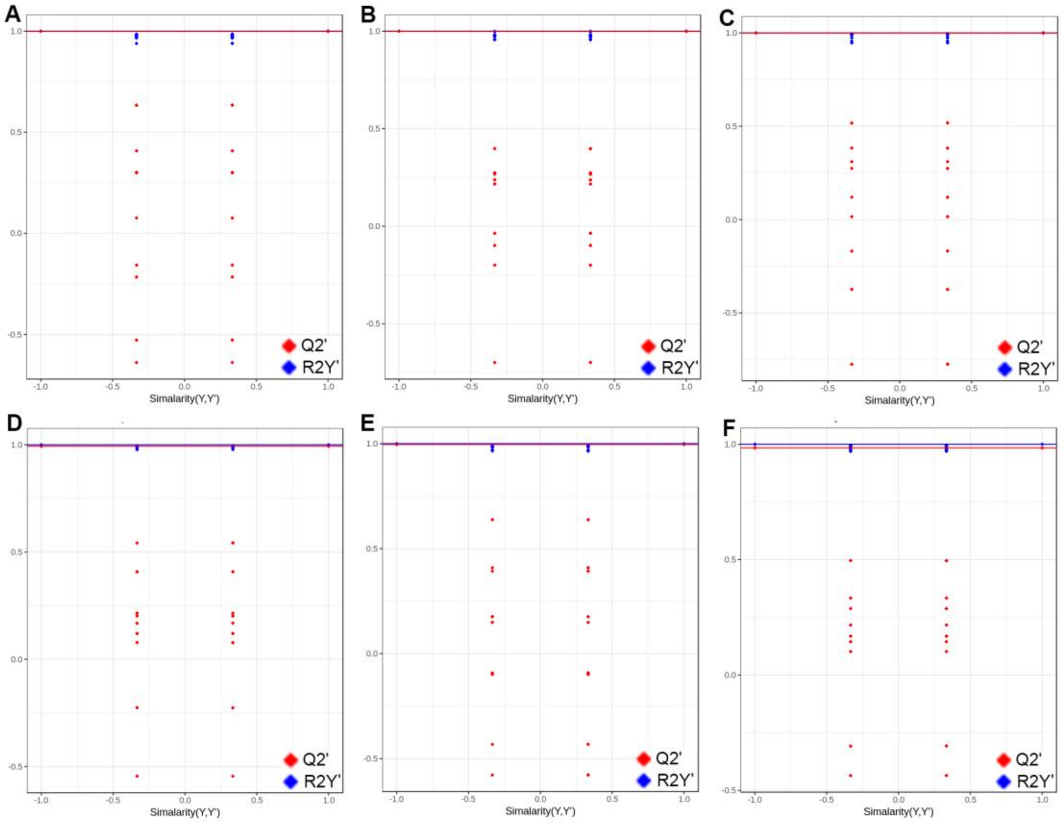

Figure S3. Permutation tests on OPLS-DA models. The horizontal line represented R2Y and Q2 calculated in the original OPLS-DA model. R2′Y and Q2′ calculated by permutation tests.

Table S4. Method validation for simultaneous quantification of icariin analogues in *E. pubescens* samples.

**Analyte**	**Regression equation**	**r^2**	**Linear range (ug/ml)**	**LOD (ng/ml)**	**LOQ (ng/ml)**
epimedin A	y=2100x-6990	0.9979	0.0162-0.54	1.873	6.242
epimedin B	y=2090x-10200	0.9972	0.0278-0.555	3.918	13.059
epimedin C	y=728x-9970	0.9984	0.053-10.6	5.678	18.928
icariin	y=5410x+6710	0.9998	0.0103-0.515	0.515	1.717
baohuoside I	y=22300x+55400	0.9986	0.00515-0.386	0.2116	0.7055
baohuoside II	y=13500x+3390	0.9996	0.00129-0.645	0.2764	0.9214
sagittatoside A	y=2060x+3190	0.9995	0.0054-0.405	0.7714	2.5714
sagittatoside B	y=4100x+2630	0.9991	0.002475-0.495	0.2121	0.7071
icaritin	y=25600x+5840	0.9990	0.00107-0.0535	0.2816	0.9386
